# 0108. Patients' response to changes in ventilator support. A modelling approach

**DOI:** 10.1186/2197-425X-2-S1-P15

**Published:** 2014-09-26

**Authors:** S Larraza, N Dey, DS Karbing, M Nygaard, R Winding, SE Rees

**Affiliations:** Aalborg University, Department Health Science and Technology, Aalborg, Denmark; Regions Hospital Herning, Department of Anaesthesia and Intensive Care, Herning, Denmark

## Introduction

Understanding patients' response to changes in support ventilation is difficult. Reducing support may result in different patterns of response including changes in depth and frequency of ventilation, possible fatigue and changes in metabolism due to increased respiratory work.

## Objectives

Present and evaluate a mathematical model approach describing patients' response to changes in ventilator support.

## Methods

A set of mathematical models including pulmonary gas exchange, acid-base chemistry of blood and cerebrospinal fluid, blood oxygenation, O_2_ and CO_2_ transport and storage, ventilation, muscle function and chemoreflex respiratory drive were combined. The set of models was used to simulate changes in support during volume and pressure support ventilation. The model set was evaluated in 12 and 5 patients on volume and pressure support respectively. Ethical approval was obtained from the committee of Mid-Jutland, Denmark. Patient or relatives and general practitioners consent was obtained.

For each patient, measurement of pulmonary gas exchange was followed by a series of up to 5 step modifications in the level of ventilator support, with these steps being 50 ml, for volume support, or 2 cm H_2_O for pressure support. Each level was maintained for 15 minutes, and arterial blood gases measured. Airway pressure, flow, CO_2_ and O_2_ were measured continuously throughout the protocol.

The model set was tuned to the data of each patient at baseline conditions. Simulations of patient response at each level of ventilator support were then performed. Agreement between model simulated values and measurements of arterial pH, end tidal CO_2_ (FeCO_2_) and respiratory frequency (f) are reported as Bland-Altman bias and limits of agreement.

## Results

Volume support levels ranged from 0.26-0.65 l and pressure support from 0-14 cmH_2_O. The resulting values of respiratory frequency ranged from 10-42 min^-1^, pHa from 7.29-7.50 and FeCO_2_ from 2.2-7.7 %. The bias and limit of agreement between model predicted and measured values across all patients and at all levels of volume and pressure support were: f 0.0±1.3 min^-1^, pHa -0.001±0.03 and FeCO_2_-0.00±0.002 %.

**Figure 1:**
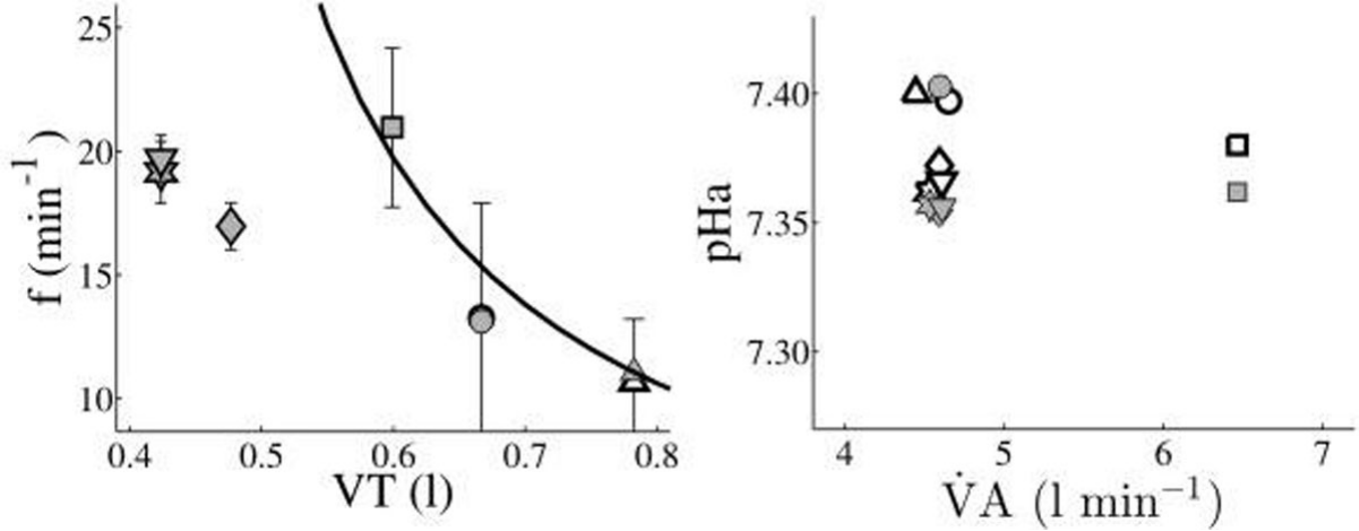
Patient response to changes in PS ventilation. Symbols represent each level of PS. Blank symbols represent measurements. Filled symbols represent model simulated values.

## Discussion

The model describes accurately patient response to changes in ventilator support, identifying the individual patient's chemoreceptor drive and situations where the patient could not respond adequately, resulting in changes in pH. The model description may help to describe patients' response to changes in ventilator support and hence optimize mechanical ventilation for the specific patient.

## References

[1] Georgopoulos D, Roussos C (1996). Eur Respir J.

[2] Duffin JJ (2005). Appl Physiol.

[3] Gilstrap D, MacIntyre N (2013). Am J Respir Crit Care Med.

